# Blood monocyte counts as a prognostic biomarker and predictor in Chinese patients with idiopathic pulmonary fibrosis

**DOI:** 10.3389/fmed.2022.955125

**Published:** 2022-11-08

**Authors:** Xinran Zhang, Yanhong Ren, Bingbing Xie, Qiao Ye, Chenjun Ban, Shu Zhang, Min Zhu, Yan Liu, Shiyao Wang, Jing Geng, Xuan He, Dingyuan Jiang, Jiarui He, Shi Shu, Sa Luo, Xin Wang, Dingyun Song, Mingming Fan, Haishuang Sun, Huaping Dai

**Affiliations:** ^1^Department of Clinical Research and Data Management, Center of Respiratory Medicine, China-Japan Friendship Hospital, Beijing, China; ^2^National Center for Respiratory Medicine, Beijing, China; ^3^Institute of Respiratory Medicine, Chinese Academy of Medical Sciences, Beijing, China; ^4^National Clinical Research Center for Respiratory Diseases, Beijing, China; ^5^Department of Pulmonary and Critical Care Medicine, Center of Respiratory Medicine, China-Japan Friendship Hospital, Beijing, China; ^6^Institute of Respiratory Medicine, Peking Union Medical College, Chinese Academy of Medical Science, Beijing, China; ^7^Department of Pulmonary and Critical Care Medicine, Beijing Chao-Yang Hospital, Capital Medical University, Beijing, China; ^8^Department of Respiration, Dongzhimen Hospital, Beijing University of Chinese Medicine, Beijing, China; ^9^Beijing University of Chinese Medicine, Beijing, China; ^10^The Second Hospital of Jilin University, Changchun, China; ^11^The First Hospital of Jilin University, Changchun, China

**Keywords:** idiopathic pulmonary fibrosis, prognosis, monocyte counts, biomarker, predict model

## Abstract

**Objectives:**

We sought to evaluate the prognostic value of blood routine parameters and biochemical parameters, especially inflammation-related biomarkers, and establish an inflammation-related prognostic model in Chinese patients with idiopathic pulmonary fibrosis (IPF).

**Material/methods:**

Patients diagnosed as IPF at Beijing Chaoyang Hospital and aged 40 years and older were consecutively enrolled from June 2000 to March 2015, and finally, a total of 377 patients were enrolled in the derivation cohort. The follow-up ended in December 2016. We used Cox proportional hazard model to calculate the hazard ratio (HR) and establish the prognostic model. The discrimination and calibration of the prognostic model were evaluated in an independent validation cohort enrolled from China-Japan Friendship Hospital between January 2015 and December 2019.

**Results:**

Multivariate analysis revealed that patients with elevated monocyte-to-red blood cell count ratio (MRR) and monocyte counts showed increased risk of mortality. The clinical-physiological-biomarker (CPB) index and CPB stage we established in this study were a significant predictor, and the C-index for CPB index and CPB stage in the validation cohort was 0.635 (95% CI: 0.558–0.712) and 0.619 (95% CI: 0.544–0.694), respectively. Patients in CPB stage III had the poorest survival.

**Conclusion:**

We developed and validated a new inflammation-related prognostic model (CPB index and CPB stage) which was integration of age, gender, FVC (%, predicted), DLCO (%, predicted), Charlson Comorbidity Index, and blood monocyte counts. This prediction model exhibited strong ability in predicting mortality in Chinese patients with IPF.

## Introduction

Idiopathic pulmonary fibrosis (IPF) is a fatal, progressive interstitial pneumonia, with median survival time being 2–3 years (1). The prevalence of IPF varies greatly from country to country; however, the incidence is rising globally (2). Patients with IPF experienced an irreversible decline of lung function. Pirfenidone and nintedanib, which are approved to treat IPF, could reduce lung function decline. The disease course is heterogeneous. The majority of patients showed a slow and gradual predictable decline in lung function, while a small number of patients experienced repeated acute exacerbations (AE), and a small number of patients showed progressive development in a short period of time (3). Therefore, it is urgent to predict the risk of mortality and stratify patients who are at risk of more rapid disease progression.

In recent years, several prognostic models have been established and widely applied in IPF, such as clinical-radiologic-physiologic scoring system, composite physiologic index, and GAP model (4–6). However, biomarkers were hardly included in these models, especially inflammation-related biomarkers, since mounting evidence has revealed that chronic inflammation may be involved in the pathogenesis and progression of IPF (7–9). Pathological analysis suggested that both innate and adaptive inflammatory cell were associated with rapid disease progression (9). Several inflammation indexes, such as monocyte, neutrophil-to-lymphocyte ratio (NLR), platelet-to-lymphocyte ratio (PLR), systemic inflammation index (SII), systemic inflammation response index (SIRI), and aggregate index of systemic inflammation (AISI), were found to associate with IPF development and prognosis (10–12). However, few prognostic models included both clinical and inflammatory biomarkers to predict mortality in Chinese patients with IPF.

Our present study aimed to evaluate the prognostic effect of blood routine parameters and biochemical parameters and identify simple and cost-effective prognostic biomarkers. Since there were various factors contributed to the development of IPF, it may be difficult to accurately predict mortality using a single biomarker. We further combined multiple indicators to predict mortality in patients with IPF.

## Methods

### Study population

In this study, we had two IPF cohorts. Patients with IPF admitted to Beijing Chao-Yang Hospital between June 2000 and March 2015 were enrolled in the derivation cohort. The follow-up of the derivation cohort ended in December 2016. Patients with IPF admitted to China-Japan Friendship Hospital between January 2015 and December 2019 were included in the validation cohort, and the follow-up ended at August 2021. Patients who lacked HRCT results or follow-up information were excluded. All patients were re-evaluated as IPF by multi-disciplinary team, which was composed of clinical experts, imaging experts, and pathologists. The Ethics Committee of Beijing Chao-Yang Hospital and China-Japan Friendship Hospital approved the present study.

### Data collection

All data were collected during the same hospital period. The reason for hospitalization was for diagnosis or due to acute worsening of pulmonary fibrosis. Smoking status was classified into never, current, and former smoking. We collected comorbid diseases and calculated the Charlson Comorbidity Index (CCI) using Quan's method according to ICD-10 coding algorithms (14). The CCI is a weighted measure that takes all comorbid conditions present into account. Baseline spirometry data were collected using standard protocol. Blood routine parameters and biochemical parameters were measured after admission using fasting venous blood samples taken at the morning and tested within 4 h after collection. NLR, PLR, lymphocyte-to-monocyte ratio (LMR), monocyte-to-red blood cell count ratio (MRR), and albumin-to-globulin ratio (AGR) were calculated as derivations. We collected survival information from medical records or by telephone interviews.

### Statistical analysis

Continuous variables were presented as mean ± SD or median (interquartile) and compared by Student's t-test or Wilcoxon rank-sum test. Categorical variables were presented as frequency (percentage) and compared by chi-square test or Fisher's exact test. Kaplan–Meier and log-rank test were used to calculate survival rate and compare survival time. We organized the continuous variables into categorical variables according to the optimal cutoff value determined by survival tree analysis (available at the website: http://c2s2.yale.edu/software/stree/). The survival tree was developed on the basis of classification and regression tree (CART) which was proposed by Gordon and Olshen, and the researchers then improved this method. The survival tree method has a wide range of application, has no restrictions about the distribution of survival data and types of independent variables, and was widely applied to the analysis of censored survival data. By the survival tree algorithm, patients were recursively split into two groups according to many cutoff points, and the cutoff points were optimal when the two groups have the minimum *p*-value for the log-rank test. Weighted Schoenfeld residuals were used to check proportional hazard assumption, and after checking, Cox proportional hazard model was used to calculate hazard ratio (HR) and 95% confidence interval (CI).

We established an inflammation-related risk scoring system by adding monocyte counts into age, gender, FVC (%, predict), DLCO (%, predict), and CCI. We first established a prediction model using age, gender, FVC (%, predict), and DLCO (%, predict), since these indicators were composition of the GAP model which was proved to be associated with prognosis in patients with IPF. Then, CCI and monocyte counts were successively added. The point for each indicator was established as the method developed by Sullivan et al. (15). Multivariate regression model was used to estimate the regression coefficient for variables. The point for one variable equaled to its regression coefficient divided by the coefficient with the smallest absolute value in the model and rounded to the nearest integer. The total point for each participant was calculated as sum of the points for all indicators. Model performance was evaluated from discrimination and calibration. Discrimination reflects the ability to distinguish non-survivors from survivors, while calibration reflects the consistency between the probability predicted by the prognostic model and that observed. SAS software, version 9.4 (SAS Institute Inc.) and R software (version 4.0.2) were conducted to do all statistical analyses.

## Results

### Basic information of patients with IPF in the derivation cohort

We enrolled 377 patients with IPF comprising 317 male patients and 60 female patients in this study. The median follow-up time was 28.03 months, and the longest follow-up time was 168.17 months. During the follow-up time, 221 deaths occurred with the mortality and median survival time being 86.42% and 37.20 months, respectively. As shown in Table 1, the baseline percent predicted FEV1 (*p* = 0.043), percent predicted FVC (*p* = 0.025), and percent predicted DL_CO_ (*p* = 0.008) were significantly lower in non-survivors than survivors (Table 1). No significant difference was founded for blood parameters when comparing non-survivors with survivors (Supplementary Table S1).

**Table 1:** Baseline characteristics of patients with IPF in the derivation cohort.

**Characteristics**	**Non-survivors**	**Survivors**	***p***
	***N* = 221**	***N* = 156**	
Age (years)	64.51 ± 9.46	65.16 ± 9.23	0.505
Males	188 (85.07)	129 (82.69)	0.535
Smoking			
Never smoker	45 (27.44)	32 (27.12)	0.927
Current smoker	29 (17.68)	23 (19.49)	
Ever smoker	90 (54.88)	63 (53.39)	
FEV1, % predicted	74.20 ± 19.97	79.61 ± 22.16	0.043
FVC, % predicted	71.66 ± 19.91	77.39 ± 20.31	0.025
FEV1/FVC, %	82.83 ± 9.20	82.20 ± 8.90	0.582
DLCO, % predicted	33.35 ± 16.41	40.36 ± 18.71	0.008
Charlson Comorbidity Index	0.86 ± 1.12	0.90 ± 1.12	0.706

Data are expressed as median (interquartile range) or count (percentage) where appropriate. P was calculated by the t-test for continuous variables and the Chi-square test and Fisher's exact test for categorical variables.

### Risk estimates of single blood parameters and derivate parameters for mortality

Effect-size estimates of blood routine parameters, biochemical parameters, and their derivations for mortality by per standard deviation increment in the derivation cohort were shown in Table 2. After adjusted for age, gender, FVC (% predicted), DL_CO_ (% predicted), Charlson Comorbidity Index (CCI), and drug therapy (steroids, N-acetylcysteine), increased levels of monocyte (10^9^/L, HR: 1.25; 95% CI: 1.02–1.53) and MRR (1.24; 1.02–1.50) were significantly associated with increased risk of mortality.

**Table 2:** Effect-size estimates of blood routine and biochemical parameters for mortality in the derivation cohort.

**Parameters**	**Univariate**	**Model 1**	**Model 2**	**Model 3**
Single parameters				
White blood cell count (10^9^/L)	1.12 (0.99–1.27)	1.12 (0.99–1.26)	1.04 (0.83–1.31)	1.04 (0.81–1.34)
Neutrophil (10^9^/L)	1.16 (1.04–1.30)	1.16 (1.04–1.30)	1.04 (0.82–1.32)	1.04 (0.81–1.34)
Lymphocyte (10^9^/L)	0.93 (0.81–1.07)	0.93 (0.81–1.07)	0.98 (0.80–1.22)	1.03 (0.82–1.30)
Eosinophil (10^9^/L)	0.92 (0.79–1.08)	0.92 (0.79–1.07)	0.92 (0.76–1.13)	0.86 (0.69–1.07)
Monocyte (10^9^/L)	1.13 (0.96–1.34)	1.13 (0.96–1.34)	1.26 (1.03–1.55)	1.25 (1.02–1.53)
Hemoglobin (g/L)	1.03 (0.89–1.19)	1.02 (0.88–1.18)	1.04 (0.78–1.39)	1.12 (0.69–1.81)
Platelet count (10^9^/L)	1.09 (0.96–1.25)	1.10 (0.96–1.26)	1.03 (0.82–1.31)	1.03 (0.81–1.30)
Red blood cell count (10^12^/L)	0.97 (0.83–1.15)	0.96 (0.81–1.15)	0.91 (0.72–1.14)	0.86 (0.67–1.10)
CRP (mg/dL)	1.14 (0.97–1.33)	1.14 (0.98–1.34)	1.14 (0.66–1.98)	2.12 (0.64–7.10)
Total Protein (g/L)	1.08 (0.92–1.26)	1.07 (0.92–1.26)	1.05 (0.87–1.27)	1.08 (0.88–1.33)
Albumin (g/L)	0.96 (0.84–1.10)	0.95 (0.83–1.09)	0.80 (0.62–1.03)	0.82 (0.54–1.25)
Globulin (g/L)	1.10 (0.95–1.29)	1.10 (0.94–1.28)	0.91 (0.70–1.19)	1.08 (0.70–1.67)
Prealbumin (g/L)	1.02 (0.87–1.19)	1.02 (0.87–1.20)	1.07 (0.92–1.24)	1.06 (0.90–1.24)
Cholesterol (mmol/L)	1.10 (0.94–1.29)	1.10 (0.94–1.29)	1.08 (0.87–1.34)	1.09 (0.87–1.37)
H-DLC (mmol/L)	1.14 (0.97–1.35)	1.14 (0.96–1.34)	1.08 (0.88–1.34)	1.00 (0.80–1.26)
L-DLC (mmol/L)	1.10 (0.94–1.29)	1.10 (0.94–1.29)	1.12 (0.91–1.39)	1.15 (0.92–1.44)
Triglyceride (mmol/L)	0.97 (0.83–1.13)	0.95 (0.81–1.13)	0.96 (0.80–1.15)	0.98 (0.82–1.17)
LDH (U/L)	1.26 (1.07–1.48)	1.25 (1.06–1.47)	0.66 (0.43–1.00)	0.72 (0.47–1.12)
AST (U/L)	1.12 (1.00–1.26)	1.12 (0.99–1.26)	0.77 (0.41–1.44)	0.90 (0.47–1.73)
ALT (U/L)	1.11 (0.97–1.28)	1.11 (0.96–1.29)	0.86 (0.60–1.23)	0.97 (0.67–1.40)
Total bilirubin (umol/L)	1.03 (0.88–1.21)	1.03 (0.88–1.21)	1.08 (0.83–1.41)	1.11 (0.84–1.46)
Direct bilirubin (umol/L)	1.14 (0.97–1.36)	1.15 (0.97–1.36)	1.12 (0.93–1.35)	1.10 (0.90–1.34)
Indirect bilirubin (umol/L)	0.97 (0.82–1.15)	0.98 (0.82–1.16)	0.95 (0.71–1.27)	0.99 (0.73–1.33)
Derivates				
NLR	1.20 (1.06–1.37)	1.20 (1.05–1.37)	1.19 (0.78–1.82)	1.14 (0.72–1.79)
PLR	1.17 (1.04–1.31)	1.16 (1.04–1.30)	1.32 (0.96–1.83)	1.30 (0.91–1.86)
LMR	0.98 (0.82–1.16)	0.97 (0.81–1.15)	0.98 (0.79–1.21)	0.92 (0.74–1.15)
MRR	1.15 (0.97–1.36)	1.16 (0.98–1.37)	1.24 (1.02–1.51)	1.24 (1.02–1.50)
AGR	0.87 (0.75–1.00)	0.86 (0.74–0.99)	0.95 (0.74–1.22)	0.87 (0.67–1.14)

CRP, C-reactive protein; H-DLC, high-density lipoprotein; L-DLC, low-density lipoprotein; LDH, lactic dehydrogenase; ALT, alanine transaminase; AST, aspartate transaminase; NLR, neutrophil-to-lymphocyte ratio; PLR, platelet-to-lymphocyte ratio; LMR, lymphocyte-to-monocyte ratio; MRR, monocyte-to-red blood cell count ratio; AGR, albumin-to-globulin ratio; HR, hazard ratio; 95% CI, 95% confidence interval.

Effect-size estimates were calculated under the COX proportional hazard regression models for per 1 SD. Model 1 was adjusted for age and gender; model 2 was adjusted for age, gender, FVC (% predicted), and DLCO (% predicted); model 3 was adjusted for age, gender, FVC (% predicted), DLCO (% predicted), Charlson Comorbidity Index, and drug therapy (steroids, N-acetylcysteine).

### Optimal cutoff points for monocyte and MRR

**Figure 1:**
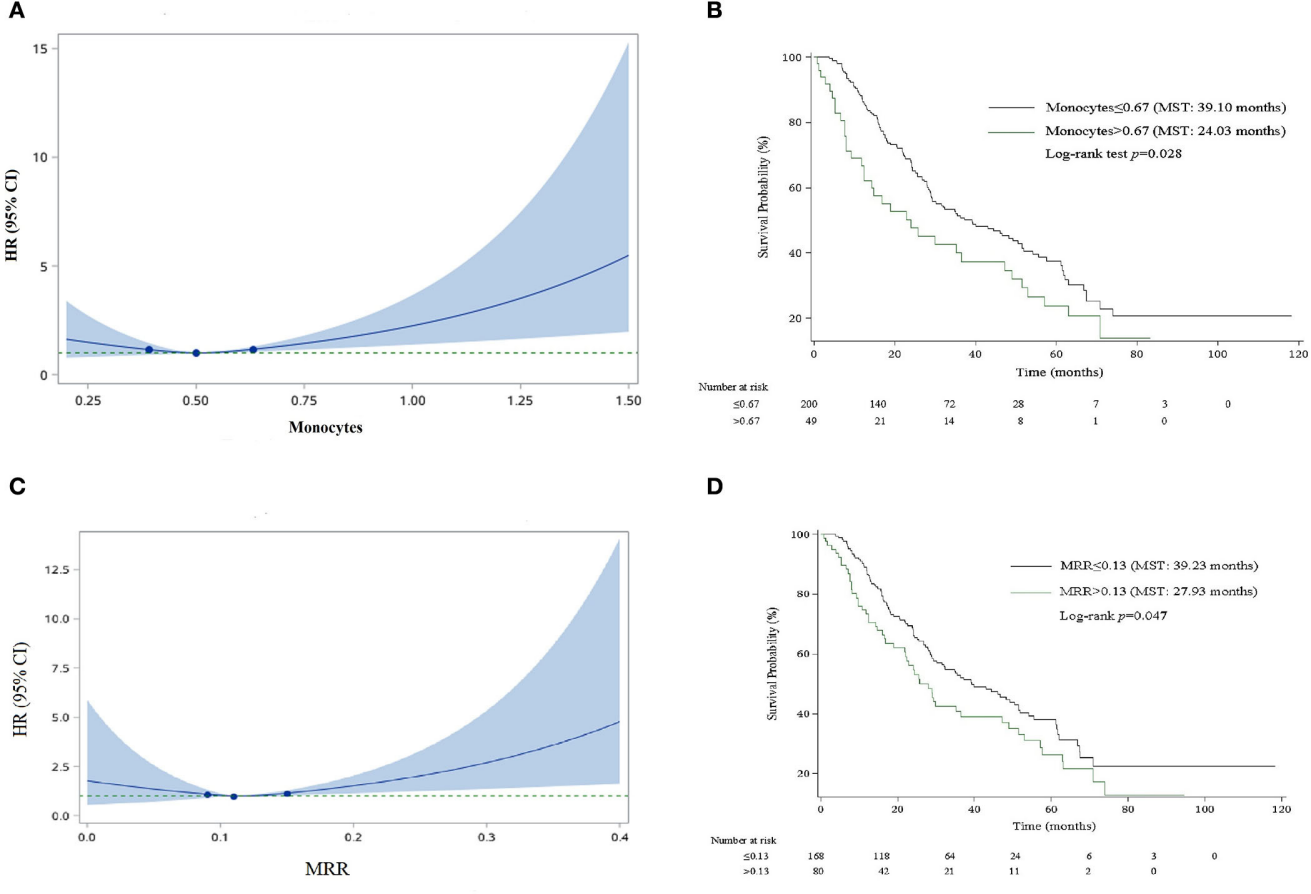
Smooth curve and Kaplan–Meier survival curves for the relationship between monocyte and MRR and risk of mortality. **(A)** Smooth curve for monocyte. **(B)** Kaplan–Meier survival curve for monocyte. **(C)** Smooth curve for MRR. **(D)** Kaplan–Meier survival curve for MRR.

For the convenience of clinical application, we organized the continuous variables into categorical variables. As shown in Figure 1, HRs for monocyte and MRR increased with the increase in the cutoff value. The optimal cutoff values for monocyte and MRR were 0.67 (10^9^/L) and 0.13, respectively. Patients with monocyte and MRR higher than the cutoff value had lower survival rate and shorter median survival time (log-rank test *p*: 0.028 for monocyte, 0.047 for MRR). In adjusted analyses, a significantly higher percentage of patients with monocyte >0.67 (10^9^/L, HR, 1.90; 95% CI, 1.16–3.11) or MRR > 0.13 (1.56; 1.02–2.37) experienced mortality (Table 3).

**Table 3:** Effect-size estimates of MRR and monocytes for mortality as categorical variable in the derivation cohort.

**Parameters**	**Case/Total**	**Survival rate, %**	**Unadjusted HR (95% CI)**	**Adjusted HR (95% CI)**
MRR				
Reference group	100/168	22.57	Reference	Reference
Risk group	53/80	12.93	1.40 (1.00–1.96)	1.56 (1.02–2.37)
Monocyte				
Reference group	119/200	20.66	Reference	Reference
Risk group	34/49	13.83	1.53 (1.04–2.24)	1.90 (1.16–3.11)

MRR, monocyte-to-red blood cell count ratio; HR, hazard ratio; 95% CI, 95% confidence interval. Effect-size estimates were calculated under the COX proportional hazard regression models.

Adjusted HR was adjusted for age, gender, FVC (% predicted), DLCO (% predicted), Charlson Comorbidity Index, and drug therapy (steroids, N-acetylcysteine). P was calculated by Kaplan–Meier analysis. Cutoff values: 0.13 for MRR, 0.67 for monocyte.

### Development of the inflammation- related risk scoring system for mortality

**Figure 2:**
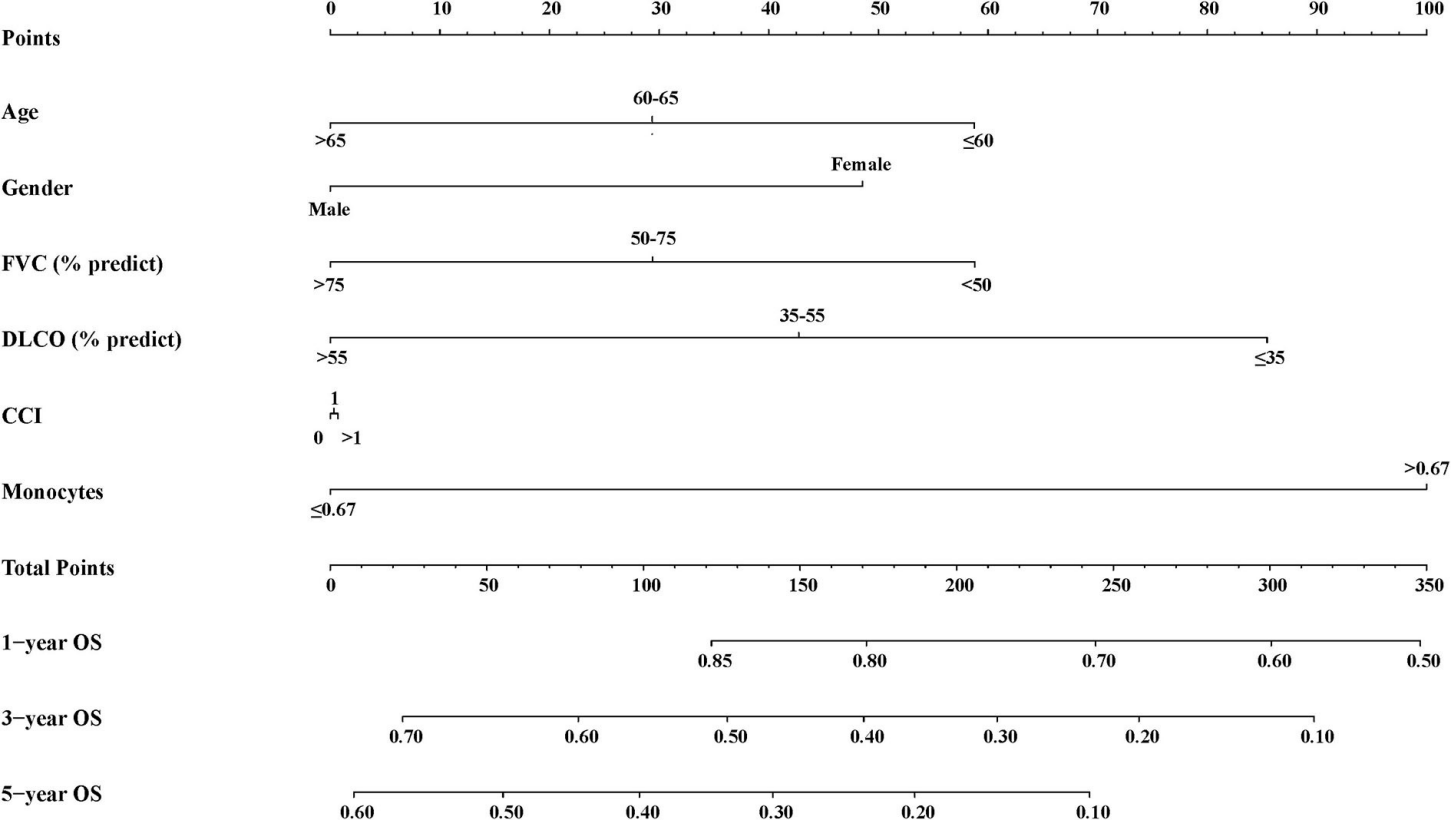
Nomogram for CPB model.

We established an inflammation-related risk scoring system by adding monocyte counts into age, gender, FVC (%, predict), DL_CO_ (%, predict), and CCI. To be more practical, we divided these indicators into categorical variables. As shown in Supplementary Table S2, the existence of six predictors in the final model performed better than other prediction models judged by the C-index, brier score, NRI, and IDI. The C-index of final model in the derivation cohort was 0.634 (95% CI: 0.576–0.693). We named the final model as the clinical-physiological-biomarker (CPB) model, and the nomogram for CPB model was shown in Figure 2.

**Figure 3:**
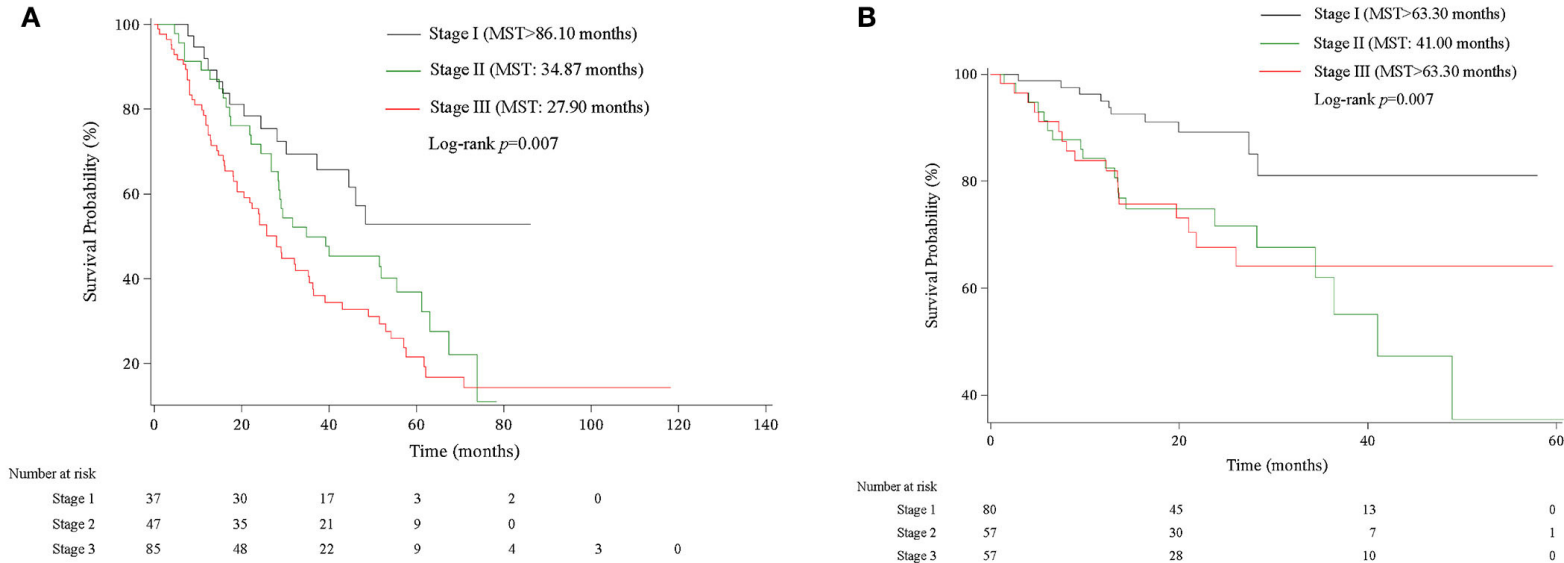
Kaplan–Meier survival curve of CPB stage in derivation and validation cohort. **(A)** Kaplan–Meier survival curve in the derivation cohort. **(B)** Kaplan–Meier survival curve in the validation cohort.

We then assigned different points for these indicators to calculate CPB index according to their regression coefficients (Supplementary Table S3). DL_CO_ (% predict) and monocyte were taken more weights in the score items. The range of CPB index was from 0 to 46 and was classified into three stages according to their distribution among all study patients in the derivation cohort (stage I: ≤ 11 point; stage II: 11–17 points; stage III: >17 points). Patients in stage III showed significantly poorer survival (log-rank *p* = 0.007) (Figure 3). Multivariable analysis revealed that patients in stage III had increased risk for mortality comparing with patients in stage I (HR: 2.22, 95%CI: 1.23–3.99) (Table 4). Predicted 1-year, 2-year, and 3-year mortality of CPB stage was closed to observed mortality in the derivation cohort (Table 5). The characteristics of patients in different CPB stage were compared (Table 6). FEV1 (% predict), FVC (% predict), FEV1/FVC, and DL_CO_ (% predict) got worse as the CPB stage increases. The percentage of current smoker was significantly higher in patients with CPB stage III. C-index for CPB index and stage in the derivation cohort was 0.632 (95% CI: 0.574–0.690) and 0.589 (95% CI: 0.537–0.642), respectively.

**Table 4:** Effect-size estimates for CPB stage in the derivation cohort.

**Variables**	**Case/Total**	**Survival rate, %**	**Unadjusted HR (95% CI)**	**Adjusted HR (95% CI)**
CPB stage				
Stage 1	15/37	52.78	Reference	Reference
Stage 2	32/47	11.05	1.71 (0.92-3.15)	1.49 (0.79-2.81)
Stage 3	62/85	14.38	2.37 (1.35-4.18)	2.22 (1.23-3.99)

Stage 1: CPB index ≤ 11; Stage 2: 11 < CPB index ≤ 17; Stage 3: CPB index > 17. Adjusted HR was adjusted for steroids and N-acetylcysteine. P-value was calculated by Kaplan–Meier analysis.

**Table 5:** Predicted and observed mortality of CPB stage in the derivation cohort.

**Mortality**	**Stage I**		**Stage II**		**Stage III**	
	**Predicted**	**Observed**	**Predicted**	**Observed**	**Predicted**	**Observed**
1-year	9.91	8.11	14.54	10.87	21.06	23.79
2-year	21.99	21.62	31.19	28.26	43.05	44.68
3-year	34.31	30.67	46.89	50.09	61.43	61.02

Stage 1: CPB index < −1; Stage 2: −1 ≤ CPB index ≤ 15; Stage 3: CPB index > 15.

**Table 6:** Characteristics of patients with IPF stratified by CPB stage in the derivation cohort.

**Characteristics**	**Stage I**	**Stage II**	**Stage III**	***p***
Age (years)	67.27 ± 6.31	68.19 ± 8.53	61.13 ± 9.54	<0.0001
Males	39 (95.12)	70 (79.55)	31 (77.50)	0.054
Smoking				
Never smoker	12 (33.33)	28 (34.15)	8 (21.05)	0.019
Current smoker	6 (16.67)	9 (10.98)	14 (36.84)	.
Ever smoker	18 (50.00)	45 (54.88)	16 (42.11)	.
FEV1, % predicted	93.87 ± 16.61	80.66 ± 16.48	69.99 ± 20.01	<0.0001
FVC, % predicted	90.39 ± 16.73	77.26 ± 16.74	66.19 ± 19.79	<0.0001
FEV1/FVC, %	81.11 ± 5.21	81.68 ± 6.66	85.46 ± 6.65	0.0003
DLCO, % predicted	52.49 ± 18.69	33.57 ± 13.52	29.55 ± 14.50	<0.0001

Data were expressed as median (interquartile range) or count (percentage) where appropriate. P-value was calculated by the Wilcoxon rank-sum test.

### External validation of the inflammation-related risk scoring system

**Figure 4:**
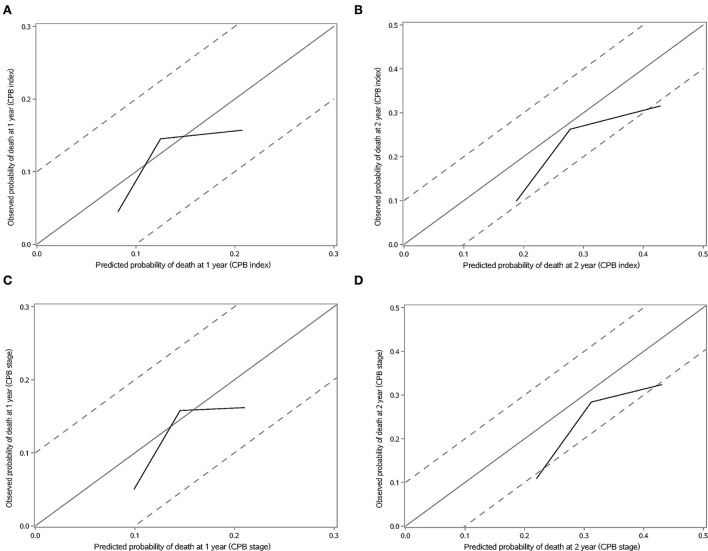
Calibration plot of CPB index and CPB stage for 1-year and 2-year mortality. **(A)** Calibration plot of CPB index for 1-year mortality. **(B)** Calibration plot of CPB index for 2-year mortality. **(C)** Calibration plot of CPB stage for 1-year mortality. **(D)** Calibration plot of CPB stage for 2-year mortality.

We validated the performance of CPB index and CPB stage by calibration and discrimination in an independent external cohort. The median follow-up time of the validation cohort was 18.23 months (range: 0.43–63.30 months). There were 75 non-survivors and 249 survivors (mortality: 39.95%) in the external cohort. As shown in Supplementary Table S4, there were significant differences for FEV1 (%, *p* = 0.006), FVC (%, *p* = 0.003), and DL_CO_ (%, *p* < 0.0001). Prognostic effect of CPB stage was first evaluated in the validation cohort, and the survival of patients was significantly poorer in stage III than stage I (log-rank *p* = 0.007) (Figure 3). Patients in CPB stage II and stage III had significantly increased risk for mortality (HR: 3.15, 95% CI: 1.47–6.75 for stage II, HR: 2.52, 95% CI: 1.13–5.62 for stage III) (Table 7). In the validation cohort, the 3-year mortality of patients in stage I, stage II, and stage III was 18.98, 38.01, and 35.93%, respectively. C-index for CPB index in external cohort and combined cohort was 0.635 (95% CI: 0.558–0.712), respectively. The discrimination for CPB stage was similar as CPB index (C-index in external cohort: 0.619, 95% CI: 0.544–0.694). Calibration for CPB index and stage was good in the derivation cohort, since the predicted 1-year and 2-year mortality by CPB stage was close to the observed mortality for CPB index and CPB stage (Figure 4). The brier score of the CPB index and CPB stage in the validation cohort for the 1-year prediction was 0.098 and 0.100, respectively.

**Table 7:** Effect-size estimates for CPB stage in the validation cohort.

**Variables**	**Case/** **Total**	**Survival** ** rate, %**	**Unadjusted** ** HR (95% CI)**	**Adjusted** ** HR (95% CI)**
CPB stage				
Stage 1	10/80	81.02	Reference	Reference
Stage 2	20/57	35.42	3.04 (1.42–6.49)	3.15 (1.47–6.75)
Stage 3	17/57	64.07	2.73 (1.25–5.96)	2.52 (1.13–5.62)

Stage 1: CPB index ≤ 11; Stage 2: 11 < CPB index ≤ 17; Stage 3: CPB index > 17. Adjusted HR was adjusted for pirfenidone, nintedanib, steroids, and N-acetylcysteine. P was calculated by Kaplan–Meier analysis.

## Discussion

In this study, we comprehensively analyzed a long-term IPF cohort and identified blood monocyte and MRR as prognostic marker in Chinese patients with IPF. We found 0.67 (10^9^/L) and 0.13 to be optimal threshold value for monocyte and MRR, respectively. Most importantly, we combined monocyte counts, age, gender, FVC (% predict), DL_CO_ (%, predict), and CCI to develop an inflammation-related risk scoring system, naming CPB index and CPB stage. The performance of the score system evaluated by calibration and discrimination was good after external validation in Chinese patients with IPF.

Significance of inflammatory biomarkers in predicting mortality in patients with IPF has been demonstrated in previous studies. In our study, monocyte, neutrophils, and lymphocyte levels were chosen to examine because they were routinely examined and more practical in clinical practice. We found a possible link between monocyte count and risk of mortality in patients with IPF, which is consistent with previous studies (16–19). Scott et al. (19) found that monocyte was a prognostic indicator independent of disease severity measured as FVC or GAP index. Those patients with monocyte more than or equal to 0.95 (10^9^/L) were considered to be high-risk population with higher mortality. On the basis of this study, Kreuter et al. conducted a retrospective analysis and found that patients with monocyte between 0.6 and 0.95 (10^9^/L) were also significantly associated with higher 1-year risk of mortality comparing with monocyte less than 0.6 (10^9^/L). The result of the Australian IPF Registry also demonstrated that elevated monocyte count was a significant predictor for poorer survival after adjusting for age, gender, and baseline FVC (% predict) (18). In our study, the optimal cutoff value for monocyte was found to be 0.67 (10^9^/L). Patients with monocyte higher than 0.67 (10^9^/L) had significantly higher mortality and shorter survival time, even after adjusted for clinical, physiological, and treatment indicators. The different cutoff values may be due to the different timing of monocyte data collection and endpoints. In addition, high monocyte level will maintain through the course of disease and may identify high-risk population earlier than other physiological indicators (20). Also, serological biomarkers which proposed previously, such as KL-6, CCL18, and MMP-7, are not available in routine clinical practice. Monocyte counts were easy to collect and minimally invasive. These findings, including ours, demonstrated that monocyte counts have great effect in predicting mortality in patients with IPF. In addition, monocyte may provide added value in predicting mortality to clinical and spirometry parameters.

Several prognostic models have been developed and widely used in IPF previously (4, 5). Among them, the GAP model developed in 2012 was the most widely used model in IPF, as well as other chronic ILD, which combined the effect of age, gender, FVC (% predict), and DL_CO_ (% predict) (4). These models were mostly a combination of clinical, radiologic, and physiological indexes. In 2017, Herazo-Maya et al. used the Scoring Algorithm for Molecular Subphenotypes (SAMS) to develop a 52-gene signature to strata high-risk patients. However, if the data were obtained by different technologies using RBA extracted from whole-blood or peripheral blood mononuclear cells, it needs to be normalized when calculating SAMS scores. The application of the 52-gene risk profile was limited. Since inflammation plays important role in IPF, several inflammatory prognostic scoring systems exist, such as GPS, SII, SIRI, and AISI. These prognostic scoring systems were different combinations of blood biomarkers. Angelo et al. found that AISI was independently associated with IPF, as well as mortality in patients with IPF (10, 11). In our study, we combined monocyte with clinical and physiological indicators to predict mortality risk in Chinese patients with IPF. These indicators were routinely provided in clinical practice. We first developed a continuous prediction model named CPB index. Each indicator was assigned the corresponding weight, and this composite model was superior to simple indexes in reflecting the status in specific disease states. Then, we classified these patients into three groups to raise the practicality of CPB index. CPB stage could separate patients into low risk, moderate risk, and high risk. We did external validation for both CPB index and CPB stage, and the performance was good when predicting short-term risk of mortality in the validation cohort. Patients with lower FVC (% predict), lower DL_CO_ (% predict), comorbidities, and higher monocyte have the worst survival.

Various immune cells were involved in the pathogenesis of IPF, such as monocytes, neutrophils, and lymphocytes (21–26). IPF pathogenesis was mainly repeated epithelial injury, aberrant repair, and the formation of fibrotic tissue (27, 28). Once the lung was injured and macrophage was depleted, monocytes will recruit to the lung and will shape into cells that closely resemble alveolar macrophages by microenvironment (29, 30). Changes in the populations of differentiated monocyte subset could lead to altered alveolar macrophage and take part in disease progression (25, 31).

NLR and PLR were derivate indicators calculated based on blood routine parameters, and the strong ability of predicting mortality was revealed for NLR and PLR in many types of diseases, especially cancer (12, 13, 32). Since IPF is similar with cancer in risk factors and pathogenesis, and inflammation plays an important role in the development and progression of IPF, therefore, NLR and PLR may play a role in predicting mortality in patients with IPF. Neutrophil count and activity were increased in IPF patients (33). Previous studies revealed that percentage of neutrophils and lymphocytes in BALF was significantly correlated with survival in patients with IPF, including ours (34). A study conducted in China found no significant association between PLR and prognosis (35). In the present study, elevated neutrophil counts, NLR, and PLR were associated with increased risk of mortality after adjusting for age and gender. However, after adjusting for more indexes, the association becomes non-significant.

Bernardinello et al. (36) found that LMR < 4.18 was associated with significantly shorter survival in newly diagnosed IPF patients. However, a significant association between LMR and mortality was not found in our present study.

There are several potential limitations in our present study. First, all-cause mortality was calculated and compared in this study because the cause of mortality was deficiency. Second, the patients included in the study belong to the same race, and it needs to be validated and adjusted when applied in other races. Third, diagnosis time in the two cohorts differs significantly due to long enrollment period, which may result to treatment difference, and we adjusted for different drug treatment in the two cohorts to lessen their effect on mortality. Fourth, the follow-up time of the validation cohort was shorter than the derivation cohort, so we validated the scoring system in predicting short-term mortality. The prediction of long-term mortality needs further validation. Fifth, the inflammatory cell profile changes at the time of acute exacerbation. The difference in the predictive power of monocytes in acute exacerbation and stable IPF will be explored in future.

## Conclusion

In conclusion, elevated monocyte counts and MRR were associated with increased risk of mortality in patients with IPF. Monocyte counts may provide a novel, simple, powerful, and cost-effective prognostic biomarker for IPF prognosis. Monocyte depletion may be a new therapy in patients with IPF in future. The prediction models named CPB index and CPB stage, which were established based on several routinely provided indicators (clinical-physiological-biomarker), were a predictor of mortality in Chinese patients with IPF. The CPB stage may help us to grade the patients. We view these findings as the beginning of future research into the role of these indicators in the progression of IPF and clues for clinical treatment.

## Data availability statement

The data are available from the corresponding author on reasonable request.

## Ethics statement

The studies involving human participants were reviewed and approved by the Ethics Committee of Beijing Chao-Yang Hospital and China-Japan Friendship Hospital. The patients/participants provided their written informed consent to participate in this study.

## Author contributions

HD planned and designed the study and directed its implementation. XZ conducted statistical analyses and wrote and revised the manuscript. YR contributed to the implementation of clinical case study, data collection, and analysis. BX, QY, CB, SZ, MZ, YL, SW, JG, XH, DJ, JH, SS, SL, XW, DS, MF, and HS contributed to data collection. All authors read and approved the final manuscript prior to submission.

## Funding

This study was supported by the National Key Technologies R&D Program (Numbers 2021YFC2500700 and 2016YFC0901101). The funders of this study had no role in study design, data collection, data analysis, data interpretation, or writing of the report. HD had full access to all the data in the study and had final responsibility for the decision to submit for publication.

## Conflict of interest

The authors declare that the research was conducted in the absence of any commercial or financial relationships that could be construed as a potential conflict of interest.

## Publisher's note

All claims expressed in this article are solely those of the authors and do not necessarily represent those of their affiliated organizations, or those of the publisher, the editors and the reviewers. Any product that may be evaluated in this article, or claim that may be made by its manufacturer, is not guaranteed or endorsed by the publisher.
