# Supplementary material for: Blood monocyte counts as a prognostic biomarker and predictor in Chinese patients with idiopathic pulmonary fibrosis
Source: Front Med (Lausanne). 2022 Nov 8;9:955125. doi: 10.3389/fmed.2022.955125 (PMC9679289; doi:10.3389/fmed.2022.955125)
Supplement: Supplementary file 1 [file Data_Sheet_1.docx]

Table S1 The differences of blood parameters between survivors and non-survivors in derivation cohort

| Characteristics | Non-survivors  N=221 | Survivors  N=156 | *p* |
| --- | --- | --- | --- |
| **Single parameters** |  |  |  |
| White blood cell count (10^9^/L) | 8.05 ± 3.32 | 7.77 ± 2.57 | 0.378 |
| Neutrophil (10^9^/L) | 5.28 ± 3.11 | 4.92 ± 2.40 | 0.236 |
| Lymphocyte (10^9^/L) | 1.93 ± 0.80 | 1.96 ± 0.77 | 0.757 |
| Eosinophil (10^9^/L) | 0.25 ± 0.22 | 0.27 ± 0.19 | 0.352 |
| Monocyte (10^9^/L) | 0.53 ± 0.24 | 0.53 ± 0.22 | 0.890 |
| Hemoglobin (g/L) | 122.10 ± 45.35 | 122.38 ± 43.31 | 0.954 |
| Platelet count (10^9^/L) | 209.01 ± 72.50 | 206.02 ± 72.55 | 0.701 |
| Red blood cell count (10^12^/L) | 4.36 ± 0.62 | 4.39 ± 0.51 | 0.649 |
| CRP (mg/dL) | 6.99 ± 21.10 | 7.09 ± 24.10 | 0.968 |
| Total Protein (g/L) | 67.96 ± 6.29 | 67.01 ± 8.47 | 0.318 |
| Albumin (g/L) | 28.52 ± 12.18 | 29.30 ± 12.20 | 0.561 |
| Globulin (g/L) | 28.99 ± 12.99 | 28.49 ± 11.97 | 0.720 |
| Prealbumin (g/L) | 0.23 ± 0.11 | 0.23 ± 0.06 | 0.767 |
| Cholesterol (mmol/L) | 4.36 ± 1.07 | 4.19 ± 0.87 | 0.219 |
| H-DLC (mmol/L) | 1.13 ± 0.33 | 1.08 ± 0.27 | 0.219 |
| L-DLC (mmol/L) | 2.61 ± 0.77 | 2.47 ± 0.67 | 0.164 |
| Triglyceride (mmol/L) | 1.31 ± 0.86 | 1.31 ± 0.84 | 0.995 |
| LDH (U/L) | 221.34 ± 84.36 | 210.65 ± 101.78 | 0.300 |
| AST (U/L) | 27.04 ± 31.89 | 24.65 ± 13.62 | 0.402 |
| ALT (U/L) | 24.34 ± 19.31 | 22.75 ± 10.95 | 0.378 |
| Total bilirubin (umol/L) | 12.21 ± 6.81 | 11.82 ± 6.07 | 0.627 |
| Direct bilirubin (umol/L) | 2.82 ± 2.67 | 2.73 ± 1.64 | 0.752 |
| Indirect bilirubin (umol/L) | 9.34 ± 5.43 | 9.23 ± 5.01 | 0.872 |
| **Derivates** |  |  |  |
| NLR | 3.50 ± 3.56 | 3.25 ± 3.61 | 0.504 |
| PLR | 132.21 ± 90.16 | 119.69 ± 68.74 | 0.156 |
| LMR | 4.17 ± 2.06 | 4.08 ± 1.79 | 0.736 |
| MRR | 0.12 ± 0.06 | 0.12 ± 0.05 | 0.616 |
| AGR | 1.04 ± 0.25 | 1.05 ± 0.24 | 0.693 |

Abbreviations: CRP, C-reactive protein; H-DLC, high-density lipoprotein; L-DLC, low density lipoprotein; LDH, lactic dehydrogenase; ALT, alanine transaminase; AST, aspartate transaminase; NLR, neutrophil-to-lymphocyte ratio; PLR, platelet-to-lymphocyte ratio; LMR, lymphocyte-to-monocyte ratio; MRR, monocyte-to-red blood cell count ratio; AGR, albumin-to-globulin ratio; Data are expressed as median (interquartile range) or count (percentage) where appropriate. *P* was calculated by the t-test for continuous variables and the Chi-square test and Fisher’s exact test for categorical variables.

Table S2 Model performance of different prediction models in derivation cohort

| Variable | C-index (95% CI) | Brier score | NRI | IDI |
| --- | --- | --- | --- | --- |
| Age + Gender + FVC + DLCO | 0.594(0.535-0.653) | 0.0959 | / | / |
| Age + Gender + FVC + DLCO + CCI | 0.598(0.541-0.656) | 0.0959 | 0.061 | 0 |
| Age + Gender + FVC + DLCO + CCI + monocyte (final model) | 0.634(0.576-0.693) | 0.0956 | 0.313 | 0.036 |

Brier score was calculated for the one-year prediction. NRI and IDI were calculated when added a new indicator into the model.

Table S3. The points for indicators of CPB index

| Indicator | HR (95% CI) | Points |
| --- | --- | --- |
| Age, y |  |  |
| ≤60 | 1.67 (0.88-3.17) | 9 |
| 61-65 | reference | 0 |
| >65 | 1.14 (0.62-2.12) | 2 |
| Gender |  |  |
| Male | reference | 0 |
| Female | 1.30 (0.79-2.15) | 5 |
| FVC, % predicted |  |  |
| >75 | reference | 0 |
| 50-75 | 1.22 (0.77-1.94) | 3 |
| <50 | 1.57 (0.78-3.14) | 8 |
| DLCO, % predicted |  |  |
| >55 | reference | 0 |
| 36-55 | 1.48 (0.67-3.29) | 7 |
| ≤35 | 1.82 (0.83-4.00) | 10 |
| CCI |  |  |
| 0 | 1.06 (0.68-1.64) | 1 |
| 1 | reference | 0 |
| >1 | 1.10 (0.57-2.12) | 2 |
| Monocyte |  |  |
| ≤0.67 | reference | 0 |
| >0.67 | 1.96 (1.21-3.17) | 12 |
| Total possible points |  | 46 |

Table S4. Comparison of baseline characteristics in derivation cohort and validation cohort

| Characteristics | Derivation cohort | | |  | Validation cohort | | | *P2* |
| --- | --- | --- | --- | --- | --- | --- | --- | --- |
|  | Non-survivors  N=221 | Survivors  N=156 | *P1* |  | Non-survivors  N=75 | Survivors  N=249 | *P1* |  |
| Age (years) | 64.51 ± 9.46 | 65.16 ± 9.23 | 0.505 |  | 67.94 ± 8.65 | 64.30 ± 8.29 | 0.008 | 0.721 |
| Males | 188 (85.07) | 129 (82.69) | 0.535 |  | 41 (87.23) | 153 (83.15) | 0.496 | 0.973 |
| Smoking |  |  |  |  |  |  |  |  |
| Never smoker | 45 (27.44) | 32 (27.12) | 0.927 |  | 10 (21.28) | 50 (26.88) | 0.106 | 0.753 |
| Current smoker | 29 (17.68) | 23 (19.49) |  |  | 4 (8.51) | 35 (18.82) | . |  |
| Ever smoker | 90 (54.88) | 63 (53.39) |  |  | 33 (70.21) | 101 (54.30) | . |  |
| FEV1, % predicted | 74.20 ± 19.97 | 79.61 ± 22.16 | 0.043 |  | 68.61 ± 18.45 | 84.92 ± 18.80 | <0.0001 | 0.006 |
| FVC, % predicted | 71.66 ± 19.91 | 77.39 ± 20.31 | 0.025 |  | 65.71 ± 19.76 | 83.03 ± 19.27 | <0.0001 | 0.003 |
| FEV1/FVC, % | 82.83 ± 9.20 | 82.20 ± 8.90 | 0.582 |  | 82.59 ± 8.56 | 81.42 ± 9.31 | 0.434 | 0.275 |
| DLCO, % predicted | 33.35 ± 16.41 | 40.36 ± 18.71 | 0.008 |  | 41.37 ± 15.74 | 53.88 ± 17.15 | <0.0001 | <0.0001 |
| Charlson Comorbidity Index | 0.86 ± 1.12 | 0.90 ± 1.12 | 0.706 |  | 1.72 ± 2.09 | 1.34 ± 1.39 | 0.1415 | <0.0001 |
| Drug therapy |  |  |  |  |  |  |  |  |
| Steriods | 33 (19.88) | 13 (11.30) | 0.056 |  | 15 (20.27) | 27 (11.54) | 0.056. | 0.202 |
| N-acetylcysteine | 127 (76.05) | 85 (73.91) | 0.683 |  | 50 (67.57) | 122 (52.14) | 0.020 | <0.0001 |
| Pirfenidone | 0 | 0 | / |  | 34 (45.95) | 130 (55.56) | 0.149 | / |
| Nintedanib | 0 | 0 | / |  | 4 (5.41) | 11 (4.70) | 0.808 | / |

Data are expressed as median (interquartile range) or count (percentage) where appropriate. *P* was calculated by the t-test for continuous variables and the Chi-square test and Fisher’s exact test for categorical variables. *P1* was the difference between non-survivors and survivors, *p2* was the difference between derivation cohort and validation cohort
